# Validity, reliability, and the contributing physical characteristics of a modified 15m prone Yo-Yo Intermittent Recovery Test Level-1 test in elite female rugby league players

**DOI:** 10.1371/journal.pone.0306171

**Published:** 2024-06-26

**Authors:** Thomas Briscoe, Josh Darrall-Jones, Omar Heyward, Ben Jones, Hayden Allen, Carlos Ramirez-Lopez, Sean Scantlebury

**Affiliations:** 1 Carnegie Applied Rugby Research (CARR) Centre, Carnegie School of Sports, Leeds Beckett University, Leeds, United Kingdom; 2 Northern Diamonds, Headingley Stadium, Leeds, United Kingdom; 3 Rugby Football Union, Twickenham, United Kingdom; 4 Premiership Rugby, London, United Kingdom; 5 England Performance Unit, Rugby Football League, Manchester, United Kingdom; 6 Faculty of Health Sciences, Department of Human Biology, Division of Physiological Sciences, University of Cape Town, Cape Town, South Africa; 7 Faculty of Health Sciences, School of Behavioural and Health Sciences, Australian Catholic University, Brisbane, QLD, Australia; 8 Scottish Rugby Union, Murrayfield, Scotland; Università degli Studi di Milano: Universita degli Studi di Milano, ITALY

## Abstract

This study aims to establish the validity and reliability of the prone Yo-YoIRL1 in elite female rugby league players (part one) and determine the anthropometric and physical characteristics contributing to 15m prone Yo-YoIRL1 performance (part two). Part one, 21 subjects completed one Yo-YoIRL1, one 20m and two 15m prone Yo-YoIRL1 tests over four sessions, with 7–14 days in-between. Part two, ten subjects completed a testing battery, including body mass, height, dual-energy x-ray absorptiometry, isometric mid-thigh pull, isometric bench-press, 10m and 20m sprints and an incremental treadmill test ($\dot{V}O_{2\max}$). The 15m prone YoYoIRL1 demonstrated poor reliability with a typical error of 68m (21%) and a smallest worthwhile change of 54m (9%). Validity analysis found the prone versions of the YoYoIRL1 were not sensitive measures of intermittent running performance. Both prone YoYoIRL1 test distances demonstrated *large* mean bias (76% and -37% respectively) and typical error of the estimate (19% and 21%, respectively) in comparison to the YoYoIRL1. Body mass (*r* = -0.89), lean mass (*r* = -0.64), body fat % (*r* = -0.68), $\dot{V}O_{2\max}$ (l∙min^-1^) (*r* = -0.64), IMTP (*r* = -0.69), IBP (*r* = -0.15), 10m (*r* = -0.77) and 20m (*r* = -0.72) momentum displayed large negative relationships with 15m prone Yo-YoIRL1 performance. Due to the poor validity of the 20m prone YoYoIRL1, the poor validity and reliability of the 15m prone YoYoIRL1, and the anthropometric and physical characteristics which negatively impact performance, practitioners should reconsider the use of the prone YoYoIRL1 test to monitor high intensity intermittent running performance.

## Introduction

Rugby league is an intermittent, collision-based team sport played internationally by men and women, at junior and senior levels [1,2]. Well-developed physical characteristics are required to optimise performance, enhance recovery, and reduce the likelihood of injury [3,4], with greater anthropometric and physical characteristics associated with a higher playing standard [5]. Therefore, the use of testing batteries to measure and longitudinally monitor anthropometric and physical characteristics is commonplace within rugby at all standards [2,6,7].

Multiple factors must be considered when designing a testing battery (e.g., cost, time to administer), with the concepts of reliability and validity two fundamental considerations when selecting which tests to include [8]. A test may be deemed valid if it accurately measures what it intends to measure, whilst a test can be considered reliable if it has a high level of repeatability, reproducibility, and consistency [9]. It should be noted that practitioners can increase the specificity of a test to enhance the test’s ecological validity, but by doing this the underlying physiological quality which is being assessed can become unclear [8].

The 20m prone Yo-Yo Intermittent Recovery Test Level 1 (20m prone Yo-YoIRL1) was developed to increase the ecological validity of the Yo-Yo Intermittent Recovery Test Level 1 (Yo-YoIRL1) for rugby league players by incorporating the action of getting up from the floor, attempting to replicate the action after a tackle during match play, at the start of each shuttle [10]. The 20m prone Yo-YoIRL1 was found to be a sufficiently reliable (TE = 66m; SWC = 48m; CV% = 9.9 and a required change = 120m) [11] and a suitable measure of rugby-specific high-intensity intermittent running (HIIR) capacity. Furthermore, the 20m prone Yo-YoIRL1 demonstrated a stronger relationship to repeated sprint speed (*r* = 0.78), fatigue index (*r* = 0.71) and mean sprint speed (*r* = 0.64) in simulated match play than the Yo-YoIRL1 [10]. However, both studies that have investigated the 20m prone Yo-YoIRL1 only used male subjects. As sex-specific differences exist in anthropometric and physical characteristics [12,13], these must be considered when identifying appropriate HIIR assessments for female rugby players.

Previous research has found males to be faster in maximal sprint testing, have greater countermovement jump heights, better agility, and have higher aerobic fitness (estimated $\dot{V}O_{2\max}$) levels when compared to female rugby players [12,13]. This is important as certain physical characteristics can influence the outcomes of other tests. For example, peak velocity over 30m has been associated with superior performance in the 30–15 intermittent fitness test [14]. By failing to account for physical differences between male and female athletes during test design, the validity and usability of a test may be negatively impacted. In the case of the 20m prone Yo-YoIRL1, the 20m shuttle distance may reduce the ability of the test to measure rugby specific HIIR in female rugby players as peak speed and the ability to change direction and accelerate become the limiting factors rather than the athlete’s HIIR capacity [12,13]. Consequently, a reduced distance (e.g., 15m) per shuttle may be more suitable for this cohort.

Therefore, the aim of this study it to determine the suitability of the 20m prone Yo-YoIRL1 in female rugby league athletes. Part one will establish the between-day reliability of the 15m prone Yo-YoIRL1 and assess the validity of the 15m and 20m prone Yo-YoIRL1 in comparison to the Yo-YoIRL1. Part two aims to investigate the anthropometric and physical characteristics which contribute to performance in the 15m prone Yo-YoIRL1.

## Methods

### Experimental approach to the problem

#### Part one

To establish the between-day reliability of the 15m prone Yo-YoIRL1 and assess the validity of the 15m and 20m prone Yo-YoIRL1 in comparison to the Yo-YoIRL1, subjects completed three variations of the Yo-YoIRL1 test, over four testing sessions, with 7–14 days’ rest between sessions. This included one Yo-YoIRL1, one 20m prone Yo-YoIRL1 and two 15m prone Yo-YoIRL1 tests. The two 15m prone Yo-YoIRL1 were conducted during consecutive sessions to assess test reliability. Reliability data was excluded if the interval was greater than 14 days to reduce the likelihood of other factors (e.g., changes in aerobic fitness) affecting the results. The Yo-YoIRL1 has been validated as a measure of HIIR in female football athletes [15] and was used as the criterion measure to assess the validity of the 15m and 20m prone Yo-YoIRL1. Subjects were asked to refrain from training in the 48 hours prior to a testing session to restrict the influence of fatigue on performance [16].

#### Part two

To assess the influence of physical characteristics on 15m prone Yo-YoIRL1 performance, a range of physical and anthropometric tests were conducted, so that relationships could be assessed. These physical and anthropometric tests were completed on day zero. Tests included body composition (body mass, height, and dual-energy x-ray absorptiometry (DEXA)), full body isometric strength (Isometric Mid-Thigh Pull (IMTP)), upper body strength (isometric bench press(IBP)), speed (10m and 20m sprints) and maximal aerobic capacity (incremental treadmill test). The 15m prone Yo-YoIRL1 was completed seven days later. Prior to body mass and body composition assessments, subjects were required to remain fasted for 10 hours. Subjects were then provided with a standardised meal before completing the IMTP, IBP, 10m and 20m sprint tests and the incremental treadmill test. Subjects were required to refrain from training for 48 hours before all testing.

#### Subjects

Recruitment for the study was split into two parts. Part one recruitment started on 25/02/2022 and finished 07/06/2022. Part two recruitment started on 06/12/2021 and finished 04/10/2022. A total of 31 elite female rugby league players participated in the study. All subjects were injury free and provided informed consent. Twenty-one female rugby league players (age = 20.5 ± 3.0 yrs, height = 163.2 ± 5.7 cm, body mass = 73.2 ± 9.1 kg) from a Women’s Super League club participated in part one of the study, and ten international female rugby league players participated in part two (age = 25.9 ± 5.5 yrs, height = 169.7 ± 5.4 cm, body mass = 75.5 ± 11.5 kg). Ethics were approved by the university’s Research Ethics Committee (Part one 95273 and part two 91130). Testing procedures were explained along with any risks and benefits to the study. Written informed consent was then obtained before any testing session.

#### Procedures

Prior to the strength, speed, and aerobic capacity tests, a standardised warm up was completed that included dynamic stretches and bodyweight movements such as squats, lunges, and push-ups.

#### Assessment of $\dot{V}O_{2\max}$

Maximal oxygen uptake ($\dot{V}O_{2\max}$) and maximum running velocity (V_max_) was determined using a running-based incremental ramp exercise test performed on a slat-belt treadmill (Woodway ELG, Woodway, Birmingham, UK). Participants completed 3 minutes of walking at 3 km/h before commencing the test at 7 km/h, increasing by 1 km/h every minute until volitional exhaustion. The treadmill was set to a 1% incline throughout the test. Pulmonary gaseous exchange was assessed using online, breath-by-breath, gas measurement (Metalyzer 3B; Cortex Medical, Germany). A two-point calibration of gas analyser was conducted in accordance with the manufacturer’s guidance. Calibration of the O_2_ and CO_2_ analyser was performed using ambient air and a calibration gas (15.00% O_2_, 5.00% CO_2_) (Cortex Medical, Germany). A 3L calibration syringe (Hans Rudolph, Kansas, USA) was used to perform a volume transducer calibration. A Bluetooth heart rate strap (Polar H10; Polar, Finland) was utilised to assess heart rate responses to exercise. The highest 30 second average $\dot{V}O_{2}$ during the incremental ramp test was used to determine $\dot{V}O_{2\max}$; V_max_ was determined as the highest running speed achieved during the incremental test.

#### Yo-Yo Intermittent Recovery Test Level 1

Subjects were required to perform 2 x 20m shuttles, starting with two feet behind the start line, interspersed with a walking recovery to a cone 5m behind the start line. At least one foot was required to touch the 20m line before changing direction and running back to the start line. The test was controlled by an audio signal with the speed increasing progressively [17]. To terminate the test, subjects could voluntarily withdraw or were removed when they twice failed to reach the finishing line in time. The last shuttle completed was recorded as the subject’s score, this shuttle was then converted to a total distance (m) by multiplying each level completed by 40 to represent the number of meters completed per shuttle. The test was performed on a grass pitch using self-selected studded footwear. The testing surface was kept consistent between testing sessions. Previous research [17] found an ICC of 0.98 and CV of 4.6% for the Yo-YoIRL1.

#### 15m and 20m Prone Yo-Yo Intermittent Recovery Test Level 1 variations

The prone 15m and 20m Yo-Yo IRL1 tests used the same audio tape as the Yo-YoIRL1. However, subjects started each stage lying prone with their head behind the start line, chest to the ground, and legs straight. Subjects were required to push themselves up before performing two shuttles interspersed with a walking recovery to a cone 5m behind the start line. The turn line was either 15m or 20m from the start line depending on which test was being performed. The 20m prone Yo-YoIRL1 has previously been shown to be reliable (CV = 9.9%) [11].

#### Body mass and height

Body mass was measured to the nearest 0.1kg using calibrated scales (SECA 213, Hamburg, Germany), with subjects wearing minimal clothing. Height was measured using a stadiometer (SECA, Hamburg, Germany) and was recorded to the nearest 0.1cm.

#### Body composition

Subjects were scanned using DEXA in line with Jones et al. (2016). During the scan, subjects wore minimal clothing whilst lying supine on the bed centralised with the relevant markers. Arms and legs were parallel to the body with a Velcro strap around the ankles to aid with support. One trained technician performed and analysed all tests according to manufacturer’s instructions. The variables used were lean mass which was recorded to the nearest 0.1kg, and body fat which was recorded to the nearest 0.1%. Previous research showed a CV of 0.5% for lean mass [2] using DEXA.

#### Isometric Mid-Thigh Pull

The IMTP was chosen as a measure of global full body strength [18]. The IMTP was performed in a specialised adjustable rack on a force plate (Kistler [family type 9260AA]). The height of the bar was required to be at the subject’s mid-thigh. Subjects completed the test twice with 3 minutes between efforts [19]. To ensure maximal performance of the test subjects were asked to pull as hard and fast as possible following a 3 second countdown [19]. The highest peak vertical force over the two attempts was used and measured in Newtons (N).

#### Isometric Bench Press

The IBP was used due to its similarity with the action required to push up off the floor at the start of each level during the prone Yo-YoIRL1. It was performed in a specialised adjustable rack on a force plate (Kistler [family type 9253B]). Elbows were required to be at 90⁰; therefore, the bar position was adjusted accordingly. Subjects were asked to press as hard and fast as possible, with 2 attempts recorded. During each attempt, verbal encouragement was provided [20]. The highest peak vertical force over the two attempts was recorded and measured in Newtons (N).

#### Speed

Speed was assessed using photocell timing gates (Brower Timing Systems, Salt Lake City, UT) over 20m, from a two-point start. Timing gates were placed at 0m, 10m and 20m. Subjects started 0.5m behind the first gates [2,21] and were instructed to set off voluntarily, exerting maximal effort to complete the sprint. Each subject had two attempts separated by three minutes rest to allow recovery. The subjects fastest time at each split distance was used for analysis, with times recorded to the nearest 0.01 seconds. Subjects performed the sprint on an outdoor grass pitch in self-selected studded footwear. An ICC of 0.91 and CV of 1.6% has previously been reported for this test’s reliability [2]. Furthermore, momentum was calculated by multiplying average split velocity and body mass together.

### Statistical analyses

All data are presented as mean ± SD, unless otherwise stated. The between-day reliability of distance ran during the prone YoYoIRL1 was quantified as the TE and coefficient of variation (CV%), alongside the SWC (0.2 x between-subject *SD*) and the minimum detectable change (MDC) at the 95% confidence level [22]. The TE was calculated using a Microsoft excel spreadsheet [23] using the following equation;

$$TE=S_{diff}∕\surd 2.$$ Eq 1

with S_diff_ as the *SD* of the difference between attempts of the prone YoYoIRL1. The MDC was calculated using the following equation as recommended by Weir (2005);

MDC=TEx1.96x√2. Eq 2

The agreement between the criterion and each version of the prone YoYoIRL1 was assessed using a freely available spreadsheet, which calculated mean bias, typical error of the estimate (TEE; prediction error for the regression equation) using the STEYX function (standard error) and Pearson correlation [24]. Both mean bias and TEE were standardized using the *SD* of the criterion measure. The standardized mean bias was rated as *trivial* (<0.2), *small* (0.20–0.59), *moderate* (0.60–1.19), *large* (1.20–1.99), *very large* (2.0–3.99) or *extremely large* (>4.0). The standardized TEE was rated as *trivial* (<0.10), *small* (0.10–0.29), *moderate* (0.30–0.59), *large* (0.60–0.99), *very large* (1.0–1.99) or *extremely large* (> 2.0). The magnitude of correlation was rated as *trivial* (<0.10), *small* (0.10–0.29), *moderate* (0.30–0.49), *large* (0.50–0.69), *very large* (0.70–0.89), or *nearly perfect* (0.90–0.99). Subjects’ best effort of the prone YoYoIRL1 was used in the validity analysis.

Pearson’s *r* was used to assess the relationship between 15m prone Yo-YoIRL1 performance and height, body mass, lean mass, body fat %, IMTP, IBP, 10 m, and 20 m sprint time and absolute and relative $\dot{V}O_{2\max}$ for part two. The coefficient of determination (*r*^2^) was used to assess the proportion of the 15m prone Yo-YoIRL1 performance that was explained by the variables measured in part two. Relative values for $\dot{V}O_{2\max}$, IMTP, and IBP were calculated by dividing the absolute value by body mass. All correlation analysis for part two was completed using SPSS version 27 (IBM, Armonk, NY, USA), with significance set at *p* < 0.05.

## Results

The mean ± SD for distance covered in Yo-YoIRL1, trial 1 and 2, and best effort of the 15m prone YoYoIRL1 and 20m prone Yo-YoIRL1 are presented in Table 1. Reliability statistics are presented in Table 2.

**Table 1:** Distances covered (mean ± SD) for Yo-YoIRL1, 15m prone Yo-YoIRL1 trial1 and 2, the subjects best 15m prone Yo-YoIRL1 and the 20m Prone Yo-YoIRL1.

	Distance ± SD (m)
Yo-YoIRL1	406.0 ± 133.8
15m Prone Yo-YoIRL1 Trial 1	585.7 ± 266.2
15m Prone Yo-YoIRL1 Trial 2	632.9 ± 285.5
Best 15m Prone Yo-YoIRL1	654.3 ± 266.6
20m Prone Yo-YoIRL1	212.5 ± 79.6

Data are mean ± SD for Yo-YoIRL1; 15m Prone Yo-YoIRL1 Trial 1; 15m Prone Yo-YoIRL1 Trial 2; Best 15m Prone Yo-YoIRL1; 20m Prone Yo-YoIRL1. Yo-YoIRL1 = Yo-Yo Intermittent Recovery Test level 1.

**Table 2:** Measures of reliability for 15m Prone Yo-YoIRL1.

	15m Prone Yo-YoIRL1
Mean Difference (90% CI) (m)	47.1 (±36.3)
Standardized mean difference	0.18 ± 0.14 *trivial*
TE (m)	68.3
CV (%)	20.8
SWC (m) (%)	53.5 (9.4)
*Small* change in performance (%) *	121.8 (30.2)
Minimum detectable change (%)	189.3 (57.7)

* The increase in distance (percentage) required to be 75% certain of improved performance of the test.

The 15 m prone YoYoIRL1 demonstrated poor reliability with a TE of 68.3 m (20.8%) and a SWC of 53.5 m (9.4%). The thresholds for 75% (SWC + TE) and greater than 95% certainty of a change (MDC) in performance in the test are 121.8 m (30.2%) and 189.3 m (57.7%), respectively.

The 15 m prone YoYoIRL1 (655.5 ± 273.4 m) demonstrated a *large* mean bias (76.3%) and TEE (19.4%) in comparison to the YoYoIRL1 (406 ± 133.8 m), with a *very large* relationship between the tests (*r* = 0.85). The 20 m prone YoYoIRL1 (212.5 ± 79.6 m) demonstrated a *large* mean bias (-37.3%) and TE (20.6%) in comparison to the YoYoIRL1 (406 m ± 133.8 m), with a *very large* relationship between the tests (*r* = 0.85) (refer to Table 3).

**Table 3:** Agreement between the criterion YoYoIRL1 and adapted prone versions of the test.

	Criterion; Yo-Yo IRL1 (m)	Practical; Prone (m)	Mean Bias % (standardized bias)	TEE % (standardized TEE)	Pearson Correlation (*r*)
**15 m Prone Yo-YoIRL1 (*n* = 20)**	406 ± 133.8	655.5 ± 273.4	76.3 ±16.6 (1.72 ±0.28; *L*)	19.4 ±1.36 (0.61 ±1.36; *L*)	0.85 ±0.12; *VL*
**20 m Prone Yo-Yo IRL1 (*n* = 16)**	400 ± 142.4	212.5 ± 79.6	-37.3 ±5.5 (-1.34 ±0.25; *L*)	20.6 ±1.42 (0.61 ±1.73; *L*)	0.85 ±0.13; *VL*

Data are mean distances covered in the 15 m and 20 m prone Yo-YoIRL1 in comparison to the criterion distance covered in the Yo-Yo IRL1 (± SD) and include percentage and standardized mean bias, typical error of the estimate, and Pearson correlation coefficient ±90% confidence intervals and descriptor; *L* = large, *VL* = very large.

Table 4 shows the relationship between anthropometric and physical characteristics with the 15 m prone YoYoIRL1. Body mass (*r* = -0.89), lean mass (*r* = -0.64), body fat percentage (*r* = -0.68), absolute $\dot{V}O_{2\max}$ (l∙min^-1^) (*r* = -0.64), IMTP peak force (*r* = -0.69), 10m and 20m momentum (*r* = -0.77 and -0.72 respectively) were all significantly correlated with 15 m prone YoYoIRL1 performance.

**Table 4:** The mean and standard deviation for anthropometric and physical characteristics of elite female rugby league players. Including the Pearson’s *r* results between 15m prone Yo-YoIRL1 and each variable.

Variable	Mean ± SD	Pearson’s *r*	Magnitude descriptor	R^2^
**15m prone Yo-YoIRL1 (m)**	921.0 ± 298.7			
**Height (cm)**	169.7 ± 5.5	-0.53	Large	0.29
**Body Mass (kg)**	75.5 ± 10.2	-0.89**	Very Large	0.79
**Lean Mass (kg)**	50.1 ± 6.0	-0.64*	Large	0.41
**Body Fat (%)**	30.1 ± 4.9	-0.68*	Large	0.46
$\dot{V}O2$ **(ml∙kg∙min**^**-1**^**)**	39.9 ± 2.8	0.59	Large	0.35
$\dot{V}O2$ **(l∙min**^**-1**^**)**	3.0 ± 0.4	-0.64*	Large	0.41
**IMTP (N)**	2207.8 ± 258.5	-0.69*	Large	0.48
**IMTP (N∙kg** ^**-1**^ **)**	29.4 ± 2.2	0.50	Large	0.25
**Isometric Bench Press (N)**	1111.7 ± 281.1	-0.15	Small	0.02
**Isometric Bench Press (N∙kg** ^**-1**^ **)**	14.8 ± 3.6	0.36	Moderate	0.13
**10 m (s)**	2.0 ± 0.08	-0.51	Large	0.26
**20 m (s)**	3.5 ± 0.2	-0.54	Large	0.29
**10 m momentum (kg** ^**-1.**^ **m** ^.^ **s** ^**-1**^ **)**	384.7 ± 49.6	-0.77**	Very Large	0.59
**20 m momentum (kg** ^**-1.**^ **m** ^.^ **s** ^**-1**^ **)**	507.1 ± 63.7	-0.72*	Very Large	0.52

Data are mean ± SD for physical testing variables. 15 m prone Yo-YoIRL1 = 15 m prone Yo-Yo Intermittent Recovery Test level 1; IMTP = Isometric Mid-Thigh Pull.

* = correlation is significant at the 0.05 level;

** = significance at the 0.01 level.

## Discussion

This is the first study to (1) determine the reliability of the 15m prone Yo-YoIRL1, (2) establish the convergent validity of the 15m and 20m prone Yo-YoIRL1 in comparison to the Yo-YoIRL1, and (3) assess the anthropometric and physical characteristics which influence 15m prone Yo-YoIRL1 performance in women’s rugby league players. Between-day reliability of the 15m prone Yo-YoIRL1 was poor with a TE and CV of 68.3 m and 20.8%, respectively, and a SWC of 53.5 m (9.4%). When assessing the agreement between the 15m and 20m prone Yo-YoIRL1 tests against the Yo-YoIRL1, mean biases and TEE were *large*, suggesting that the prone versions of the YoYoIRL1 are not representative of the YoYoIRL1, whereas the Pearson *r* (*very large*) demonstrated that there was still a relationship between the tests. Body mass, lean mass, body fat percentage, absolute $\dot{V}O_{2\max}$, IMTP, 10m and 20m momentum displayed *large* to *very large* negative relationships with 15m prone Yo-YoIRL1 performance. Overall, the *large* to *very large* negative relationships between physical characteristics and test outcomes, alongside the poor between-day reliability suggest that the 15m prone Yo-Yo IRL1 should not be used as a measure of HIIR performance in female rugby league players.

Reliability is an important factor when choosing a test for monitoring and ranking a squad’s performance. It allows practitioners to confidently state an improvement has occurred in a test [8]. Between-day reliability of the 15m prone YoYoIRL1 was poor with a TE and CV of 68.3 m and 20.8%, respectively, and a SWC of 53.5 m (9.4%). For a practitioner to be confident of a *small* change in performance an increase or decrease in distance of 122m (equivalent to five shuttles) would be required. When assessing the agreement between the 15m and 20m prone Yo-YoIRL1 tests against the Yo-YoIRL1, mean biases and TEE were *large*, suggesting that the prone versions of the YoYoIRL1 are not representative of the YoYoIRL1, whereas the Pearson *r* (*very large*) demonstrated that there was still a relationship between the tests. Therefore, practitioners should reconsider the use of the prone YoYoIRL1 variations as a measure of HIIR in female rugby league, as important changes in HIIR may be masked by other influencing factors during the test.

When incorporating a test into a testing battery it is important to understand the physiological constructs it is measuring. Large significant negative relationships were found between 15m prone Yo-Yo IRL1 performance and lean mass, body fat %, VO _2max_ (l∙min^-1^), IMTP, 10m and 20m momentum. Despite this, when $\dot{V}O_{2\max}$, IMTP, and IBP scores were made relative to body weight, *large* ($\dot{V}O_{2\max}$, IMTP) and *small* (IBP) non-significant positive relationships were identified (Table 4). This change in direction and significance may be due to the *very large* negative relationship between body mass and 15m prone Yo-YoIRL1 performance. The negative relationship between body mass and HIIR performance has been highlighted in earlier work in male rugby union and league [25,26], and it appears this finding is substantiated in female rugby league. This relationship with body mass could also be exaggerated by the prone start position of the test, as it requires subjects to get off the ground and would naturally favour players with a lower body mass who would have to complete less mechanical work in this phase of the shuttle [27–29]. However, despite the negative correlation with HIIR performance, practitioners should not look to reduce body mass in pursuit of improved test results, as this may negatively impact match performance by reducing an individual’s momentum which may reduce their effectiveness in collision situations [30]. This is an important consideration as the collision (i.e., the tackle) is the biggest injury mechanism in rugby league [31]. Therefore, a balance must be obtained between body mass and HIIR performance and testing batteries should incorporate tests which can effectively monitor both constructs.

While this study is the first to investigate the validity and reliability of the prone 15m Yo-YoIRL1, there are limitations. Firstly, it should be acknowledged that this study has a small sample size which may have impacted the certainty surrounding the results of this study. However, recruiting a larger sample size is a challenge when investigating international level athletes. Additionally, this study required participants to complete multiple in-season fitness tests. Rugby league teams typically have a periodised training structure and the incorporation of four fitness tests across weeks of training will impact pre-planned training loads. Therefore, due to the playing standard of the participants and the nature of the study design increasing the sample size was not possible. Second, players were asked to refrain from training for 48 hours before each testing session. However, it was not possible to robustly monitor the training load and recovery levels of subjects prior to each testing session due to contextual constraints (e.g., a lack of monitoring systems). Therefore, players may have participated in testing sessions with increased fatigue which may have impacted their test performance.

## Conclusions

The between-day reliability of the 15m prone YoYoIRL1 and the validity of both prone versions (15m and 20m) is poor and neither test should be used to quantify and monitor HIIR performance in female rugby league players. Body mass, lean mass, body fat %, $\dot{V}O_{2\max}$ (l∙min^-1^), IMTP, 10m & 20m momentum have very large–large negative relationships with 15m prone Yo-YoIRL1 performance. Therefore, practitioners should be aware of the multiple anthropometric and physiological characteristics which impact performance in the 15m prone YoYoIRL1 test. This is an important consideration as the anthropometric and physiological capacities that a test quantifies should be well understood before the test is incorporated into a testing battery [8].

### Practical applications

To justify the inclusion of any test within a testing battery the test needs to be a valid and reliable measure. The results from this study found that both the 15m and 20m prone Yo-YoIRL1 tests show poor validity when compared to the Yo-YoIRL1_,_ with the former also showing poor reliability. Therefore, practitioners cannot reliably use the 15m YoYoIRL1 test to monitor or measure HIIR performance in female rugby league players. Based on this evidence it is suggested that practitioners use another measure of HIIT (e.g., the 30–15 intermittent fitness test [32]) which has previously been validated in female soccer [33] to monitor performance.

## Supporting information

S1 Raw data (XLSX)
